# Author Correction: Signatures of Plexcitonic States in Molecular Electroluminescence

**DOI:** 10.1038/s41598-018-35951-x

**Published:** 2018-11-29

**Authors:** Justin P. Bergfield, Joshua R. Hendrickson

**Affiliations:** 1 Department of Physics, Illinois State University, Moulton Hall 311, Normal, IL 61790 USA; 2 Department of Chemistry, Illinois State University, Julian Hall 214, Normal, IL 61790 USA; 3 Air Force Research Laboratory, Sensors Directorate, Wright-Patterson Air Force Base, Ohio, 45433 USA

Correction to: *Scientific Reports* 10.1038/s41598-018-19382-2, published online 02 February 2018

In the original version of this Article, there were repeated typographical errors in which the words “plexciton” and “plexcitonic” were misspelled as “plexiton” and “plexitonic” respectively.

In addition, the Supporting Information file which accompanied the original Article was corrupted.

These errors have now been corrected in the HTML and PDF versions of this Article, and in the accompanying Supporting Information.

