# Supplementary material for: Signatures of Plexcitonic States in Molecular Electroluminescence
Source: Sci Rep. 2018 Feb 2;8:2314. doi: 10.1038/s41598-018-19382-2 (PMC5797164; doi:10.1038/s41598-018-19382-2)
Supplement: Supplementary file 1 — Supporting Information [file 41598_2018_19382_MOESM1_ESM.pdf]

Supporting Information: Signatures of Plexcitonic States in Molecular Electroluminescence

Justin P. Bergfield*,† and Joshua R. Hendrickson¶

†Department of Physics, Illinois State University, Normal, IL

‡Department of Chemistry, Illinois State University, Normal, IL

¶Air Force Research Laboratory, Sensors Directorate, Wright-Patterson Air Force Base, Ohio 45433, USA

E-mail: jpbergf@ilstu.edu

Plexitonic Coupling Values

The Rabi splitting of the plexcitonic states is given by $g_{ij} = F_{ij} g_0$,

$$\hbar g_0 = E_{\rm vac} \mu_e u_j(x_0), \tag{1}$$

where the vacuum field is given by

$$E_{\rm vac} = \left(\frac{\hbar\omega}{2\varepsilon_0 V}\right)^{1/2}. \tag{2}$$

Density functional calculations using the B3LYP functional predict the dipole moment of the $Q(0,0)$ transition of TPP to be μ_e =0.44eÅ.[1] Realistic values for V_0 vary significantly; for instance, a planar cavity with $\sim 1\mu m$ spacing gives $V_0 \sim 10^{-15} m^3$, while microstructures have demonstrated $V_0 \propto (\lambda/n)^3$. If we use λ=656nm (1.89eV) then $V_0 \sim 10^{-19} m^3$ for $n = 1.0$. With the vacuum field only (i.e. $E_{\rm vac}$=4.13×10^6 V/m and $u_j(x_0) = 1$), this gives $\hbar g_0$ ∼183μeV and using the Franck-Condon approximation discussed in the text $\hbar g_{11} = 0.54 g_0 = 99\mu$eV. This gives a vacuum Rabi splitting of ∼197μeV.

Using the experimental linewidths as a rough estimate, we find $\hbar\kappa \sim$50meV. The strong coupling regime can be identified from the EL when $|g|/\kappa > 0.25$. In terms of the plexcitonic coupling, this requirement for the TPP junction equates to $\hbar g_{11} > 23$meV. Therefore, the strong coupling regime can be observed with our assumed parameters when the plasmon field strength exceeds the vacuum field by $\sim$232 times or $|E_{\text{plas}}| >\sim 9.6 \times 10^8$ V/m.

STML from TPP at T=300K

Temperature plays a significant role in the EL of the TPP junction. The simulated STML for a TPP junction operating at room temperature are shown in Fig. 1.

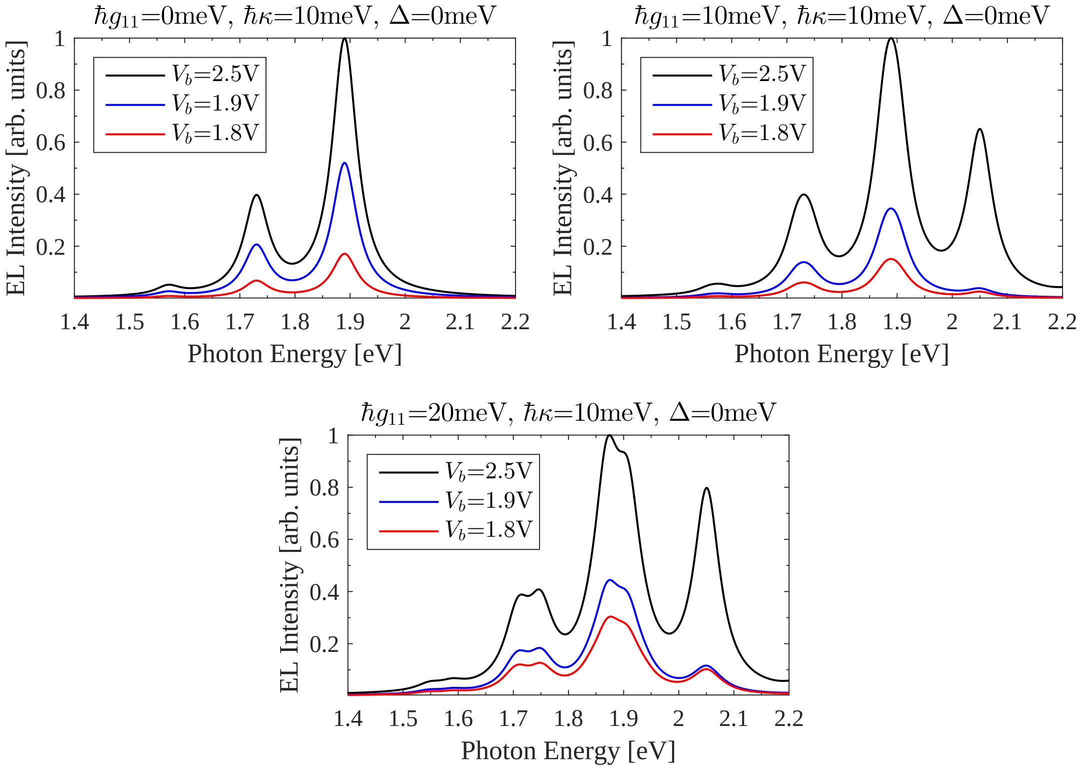

Figure 1: The calculated STML of a TPP molecule coupled to a single plasmon mode, shown as a function of photon energy for three plexcitonic coupling strengths $\hbar g_{11}$ =0meV, 20meV, and 30meV at three bias voltages V_b=1.8V, 1.9V, 2.5V. All results are for junctions operating at T=300K. In the decoupled cavity limit ($\hbar g_{11} = 0$), the molecular vibrational spectrum is recovered. The 1.8V EL is significantly enhanced as compared with the T=80K spectra

Franck-Condon Factors

The Franck-Condon factor is square of the overlap integral between nuclear wavefunctions,

$$F_{ij} = \langle i|j\rangle^2. \tag{3}$$

In this work we assume a harmonic potential landscape such that the overlap integral may be written as[2]

$$\langle i|j\rangle = \sqrt{\frac{i!}{j!}}\,(-S)^{\frac{(j-i)}{2}} \exp\left(-\frac{S}{2}\right) L_i^{j-i}(S) \tag{4}$$

where $L_m^n(x)$ are the associated Laguerre polynomials,

$$L_m^n(x) = \sum_{k=0}^{m} \frac{(-1)^k (m+n)!}{(m-k)!(n+k)!k!} x^k, \tag{5}$$

and S is the Huang-Rhys parameter.

References

(1) Tian, G.; Luo, Y. Electroluminescence of molecules in a scanning tunneling microscope: role of tunneling electrons and surface plasmons. *Phys. Rev. B* **2011**, *84*, 205419.

(2) Keil, T. H. Shapes of impurity absorption bands in solids. *Physical Review* **1965**, *140*, A601.
